# The Effect of Vacuum Forming on the Quality of Refractory Materials

**DOI:** 10.3390/ma16237260

**Published:** 2023-11-21

**Authors:** Marcin Brzeziński, Mariusz Łucarz, Alicja Trela, Alena Pribulova, Peter Futáš

**Affiliations:** 1 Faculty of Foundry Engineering, AGH University of Krakow, Reymonta 23 St., 30-059 Krakow, Poland; eumar@agh.edu.pl (M.Ł.); atp@agh.edu.pl (A.T.); 2 Faculty of Materials, Metallurgy and Recycling, Technical University of Kocice, Letna 1/9, 042 00 Kosice, Slovakia; alena.pribulova@tuke.sk (A.P.); peter.futas@tuke.sk (P.F.)

**Keywords:** melting furnace, refractory materials, innovative methods, mercury porosimetry, slag resistance

## Abstract

Various designs of furnaces for melting alloys are used in the foundry industry. Regardless of their design, they have one common detail, which is the lining of their interiors with refractory materials. This component in the design of a metal-melting furnace has a very important task—to protect the rest of the furnace assemblies from thermal and mechanical damage. Continuous technical progress and the quality requirements of casting production produce increasingly higher demands for refractory materials in connection with their development as well. The article presents the results of an innovative method of vibratory compaction of refractory material (high-alumina aluminosilicate) using reduced pressure. The analysis presents a comparative study of two methods used for forming refractory materials, i.e., the application of the mentioned innovative method and the classical (standard) method of compaction by vibration only. The effects of the introduced modification in the manufacture of ceramic shapes were evaluated by means of the material’s resistance to thermal shock, linear expansion, and dimensional change due to firing, apparent density, open porosity, and apparent specific gravity, determination of total pore volume and pore size distribution by mercury porosimetry, and slag resistance. The tests performed indicate that the procedure of lowering the pressure during the vibratory compaction of the refractory material creates a more homogeneous structure with a smaller number and size of pores. This makes it possible to improve most of the parameters that determine the quality of the refractories used for the linings of the foundry furnace.

## 1. Introduction

The foundry industry is one of the most important industries that faces increasingly difficult challenges [1,2]. Products in this industry are not only characterised by performance or aesthetics but also are often responsible for the safety of their users [3]. In most cases, casting is the basis for the creation of machine parts and assemblies. It is the starting point in many industrial fields. Therefore, companies are directing more financial resources to discover new alloys and specific ways of casting them and for creating new design solutions for equipment or manufacturing technologies to meet the industry requirements [1]. An additional goal, which cannot be ignored, is to strive to improve the quality of casting products and the productivity of their manufacturing. A high degree of complexity in the production of a product, such as a casting, stands in the way of achieving this goal. The large number and variability of parameters affecting its quality cause enormous difficulties in optimising the casting process and thus reducing the number of defects in the material. Finding the optimal conditions to achieve the minimum casting defects is very important [3].

One of the most important stages of casting production is the proper preparation of liquid metal. This is a difficult process, often consisting of several stages with high physical and thermal variability of the used operations. The final quality of the finished casting depends on the appropriate parameters of the finished alloy [1,3]. Depending on the melting technology, different furnace designs are used in the foundry industry. However, they have one common element. This is the furnace lining made of a refractory material [4]. Continuous technical progress is increasing the requirements for refractory materials as well. This component of the design of a metal-melting furnace has a very important task, to protect the other elements of the furnace from thermal and mechanical damage [5]. The alloy-melting furnace operates at high temperatures; thus, it is exposed to numerous erosive factors. Therefore, furnaces are expected to be used for a long time, without the need for costly repairs and overhauls. To ensure the elimination of any undesirable impurities in the casting melt, it is essential that the refractory coating of the furnace is absolutely not a source of these impurities [4].

Refractory materials are mainly high-alumina aluminosilicates characterised by high alumina (III) content, above 45%. They can be produced from natural raw materials and semifinished products. Natural raw materials include hydrated forms of aluminium oxide, such as hydragilite Al_2_O_3_ 3H_2_O, boehmite Al_2_O∙H_2_O, and diaspore Al_2_O_3_∙H_2_O, and aluminium (III) silicates such as andalusite, sillimanite, cyanite with anhydrous oxide with formula Al_2_O_3_ SiO_2_, and the less common, dumortierite 8Al_2_O_3_ 6SiO_2_B_2_O_3_ H_2_O or topaz Al_2_(F,OH)_2_SiO_4_. Intermediates used in the production of high-alumina materials include technical alumina (III), fused and sintered alumina, or mullite fingering [4,6].

A higher alumina (III) content makes it possible to obtain products with better strength and refractory properties, but natural raw materials are usually contaminated with harmful ferrous or calcareous admixtures, necessitating the use of enrichment and purification of raw materials. Failure to remove impurities can cause a drastic decrease in thermal properties [4].

The study in [7] provides an overview of applications and a historical perspective on the use of silica and alumina-based refractories. 

Refractory materials are widely used where high heat resistance and the lack of interaction with liquid metal are needed. The high demands placed on them by the foundry industry entail a number of parameters [4,8].

The porosity of the refractories is the most important parameter. It is directly related to their apparent density, which determines the occurrence of erosion of the furnace lining due to the casting alloys and the slag formed in the smelting process. The porosity of the material also affects mechanical properties: compressive or bending strengths. A distinction is made between open and closed porosity. Open pores have a great influence on the dynamics of slag penetration into the refractory material; thus, this porosity should be as low as possible. A larger volume of open pores results in a more intense penetration of liquid slag deep into the furnace lining, which affects the wear rate. Therefore, a better solution, which is being pursued, is to increase the number of small pores compared to the number of large pores [4,9,10].

The mechanical properties of refractory materials determine the strength of the finished products toward external and internal stresses [11]. The parameter studied is the compressive strength, i.e., CCS (Cold Crushing Strength). Refractory materials used, for example, in the lining of blast furnaces are mainly subjected to compressive stresses [11]. The lower the compressive strength, the greater the chance that the furnace lining will crack in the event of a misfiring [12].

The thermal properties of refractory materials are very important because of their operation at high temperatures. The primary thermal property determined is the thermal expansion, which directly affects the multiplicity of expansion joints.

Classical parameters for assessing the quality of refractory materials, such as the evaluation of compressive strength and porosity using optical analysis, are widely available and are therefore quite often used in industry [13,14,15]. Their analysis in most cases is sufficient for ongoing quality control both during the production of these materials and for verification during operation under industrial conditions. However, in most cases, in addition to the basic aspects and industrial assessments, the methods used in laboratories provide important additional information that helps in the selection of refractory materials, the determination of damage parameters, the interpretation of damage based on theoretical data, and the understanding of mechanisms. Through these studies, the optimisation of the manufacturing process of refractory materials and cladding design can be proposed. Knowing the properties of refractory materials involves the availability of a full range of resources for laboratory analysis and requires in-depth expertise. 

Advanced laboratory testing using a mercury porosimetry [12,16], which takes into account the analysis of parameters such as apparent density, pore volume, the distribution of a refractory material, and resistance to penetration of slag, can help verify the quality of manufacturing methods in the search for high-quality products.

Mercury porosimetry is one of the basic methods for determining the pore structure parameters of porous materials. With its help, it is possible to determine the actual porosity of the material under study, the distribution of the pore size and surface area, and the specific surface area [17,18,19,20]. The principle of mercury porosimetry that relates pressure to pore size was first established by Washburn [12]. The method is based on the experimental determination of the so-called capillary potential curve, which relates the volume of mercury pressed into a porous material to the pressing pressure, and its interpretation based on a model of the course of this process in the porous material [16,18,21].

Refractory materials, commonly used in the foundry industry as linings of furnaces used for melting metal alloys, are the first and only protective boundary that protects the remaining furnace components from liquid metal. Due to their resistance to high temperatures and erosion in the work environment, they are the best solution in this regard. However, because of the very harsh conditions they are subjected to, their service life is limited. Erosion is mainly caused by slag and molten metal and is the primary phenomenon responsible for the degradation and consequent wear of refractory bricks. It is a complex phenomenon that results from infiltration (penetration) into and subsequent dissolution of the refractory material by slag and molten metal [15,22,23,24,25]. Reducing the porosity of a refractory material can help increase its resistance to the adverse effects of slag, thus increasing its service life and reducing waste formation [5].

The resistance to thermal shock often determines the choice of refractory material when it is exposed to significant temperature gradients during its use. This includes both continuous and periodic high-temperature processes [22]. Thermal shock, i.e., an abrupt change in temperature, causes thermal stress in the material. If this stress exceeds the critical value of the material, it will cause the initiation and subsequent propagation of cracks, resulting in the failure of the refractory material [22,26].

Refractory materials have a certain thermal expansion, which depends on composition and temperature, among other factors [23]. This is a very important parameter from the point of view of the construction of a furnace lining for the melting of metal alloys. The expansion must be taken into account during design by adopting a certain dilatation between the individual cubes. An additional important parameter of the refractory material is its permanent change during heating.

As noted in the study of [27], currently no laboratory test can reproduce the actual conditions in the working steel industry, which does not change the fact that research on mitigating the effects of metal and slag erosion on refractory materials is not being carried out. Laboratory tests involve analysing the complexity of many related phenomena (thermal properties, chemistry, mechanics, etc.) and implementing them at high temperatures, which generate additional difficulties during experiments.

The high requirements lead to a constant search for new and better modern methods for obtaining finished refractory products. One such solution is the use of reduced pressure when forming refractory shapes.

The use of reduced air pressure as a method for compacting bulk materials is well known [28,29]. In the paper [30], the design and operating parameters of green sand compactors used in foundry applications were analysed. The results of experimental and simulation studies were used to analyse and evaluate the moulding sand compaction process. 

The analysis of this concept was one of the sources of such an attempt to compact ceramic materials.

## 2. Materials and Methods

### 2.1. Materials

#### 2.1.1. Refractory Materials

The study was carried out on the most commonly used aluminosilicate refractory materials in the foundry industry [3,4]. Aluminosilicate materials, according to the Polish standard PN-EN 993-12:2000 [6], consist of two main components, SiO_2_ and Al_2_O_3_, in different proportions. High-alumina aluminosilicate was used in this study.

#### 2.1.2. Slag

- Slag was prepared for slag resistance testing. Two sets of proportional slags were prepared and are as follows:13.5 g of steel mill ladle slag and 1.5 g of CaF_2_;
- 13.5 g of synthetic slag and 1.5 g of CaF_2_.

### 2.2. How to Prepare Refractory Materials for Testing

The refractory material was made in a ZYKLOS mixer. A 75 kg mass of finished refractory material was prepared by mixing the dry ingredients with water until a moisture content of 6% was achieved. 

The mixing was carried out according to the following scheme:

- Mixing 70.5 kg of dry mass (temperature 23.3 °C) for 1 min;
- Adding 4.5 kg of water (temperature 18.3 °C) and mixing for 10 min.

The temperature of the finished mixture was measured, which was 27.5 °C.

After mixing the material, two blocks were formed by the following means:

- by vibrating at atmospheric pressure (STD);
- by vibrating with a vacuum pump (VAC), creating a pressure of 4.9 kPa in the mould.

**Figure 1:**
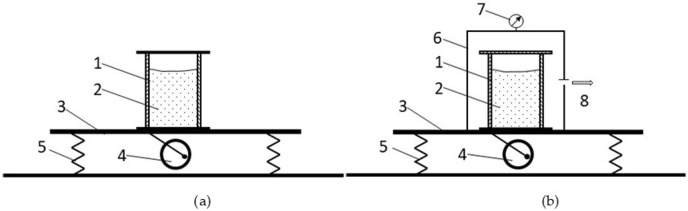
Diagram of the compaction of moulds with refractory material: (**a**) by vibration at atmospheric pressure (STD) and (**b**) by vibration with a vacuum pump (VAC) (1—mould box, 2—refractory materials, 3—vibrating table, 4—eccentric mass, 5—spring, 6—vacuum case, 7—vacuum gauge, and 8—vacuum pump).

**Figure 2:**
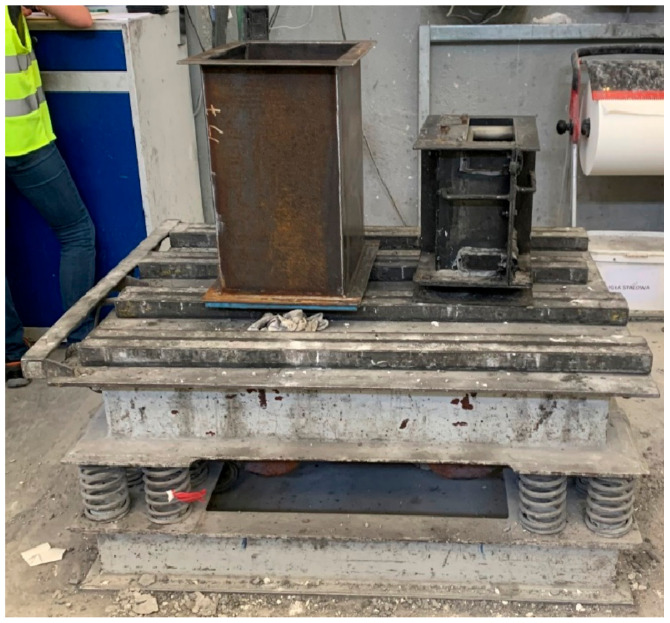
Moulds on a vibrating table [31].

**Figure 3:**
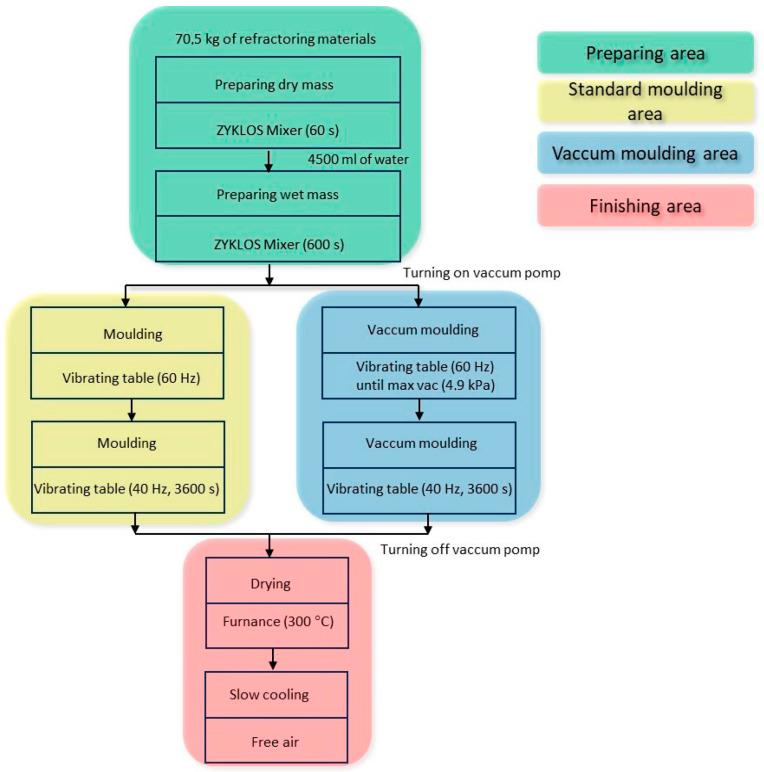
Flow chart of material for testing.

Two identical unfolded metal moulds, lubricated from the inside with a release agent to facilitate removal after drying, were prepared for the tests. Once the moulds were assembled, one of them was placed in a special sealed enclosure, and a vacuum pump and vacuum gauge were connected to it. Both moulds were compacted on a vibrating table at a vibration frequency of 60 Hz. After the mould pressure of 4.9 kPa was reached, the vibration frequency of the vibrating table was reduced to 40 Hz, and the mass was compacted for 8 min. Figure 1 shows the idea of shaping, while Figure 2 shows the experimental stand. The fabrication of the test materials is also shown in Figure 3.

### 2.3. Methods

The suitability of vacuum formation as an alternative to classical compaction was assessed by analysing the prepared specimens using several types of tests:

- Testing for resistance to sudden temperature changes (thermal shock resistance).
- Determination of permanent dimensional change due to heating to a certain temperature.
- Determination of the change in length due to heat.
- Determination of apparent density, open porosity, and apparent specific gravity.
- Determination of the effect of temperature on the dimensional change of the samples.
- Determination of the total pore volume and pore size distribution using mercury porosimetry.
- Testing of slag resistance.

#### 2.3.1. Thermal Shock Resistance Test

For the thermal shock resistance test, 16 samples measuring 20 × 20 × 80 mm ± 0.5 mm were prepared—8 cut from a block moulded at atmospheric pressure (STD) and 8 cut from a block moulded under reduced pressure (VAC). From each moulding set, 5 samples were dried and 3 were fired.

The samples were dried in a laboratory dryer at 110 ± 5 °C to a constant weight and labelled from (STD1) to (STD5) and from (VAC1) to (VAC5). The remaining samples were fired in a laboratory oven at 1600 °C according to the following procedure:

- Heating to 1200 °C for 4 h.
- Heating to 1550 °C for 2 h and 55 min.
- Heating at 1600 °C for 50 min and holding for 5 h.
- Cooling to 100 °C for 5 h.

After firing and cooling, the samples were labelled from (STD1B) to (STD3B) and from (VAC1B) to (VAC3B), respectively. Then, after heating the oven to 1200 ± 50 °C, all shapes, except the two fired ones (STD1B and VAC1B) and the two dried ones (STD1 and VAC1), were placed in the oven and held for 15 min. After this time, the samples were removed and dropped into a tank of running water at 20 ± 2 °C for 3 min. The samples were removed from the water, and any damage was visually assessed. The samples were then dried in a laboratory dryer at 110 ± 5 °C for 30 min. Each dried sample was set aside before the next cycle, which consisted of heating and rapid cooling (STD2 and VAC2). No fired samples were kept. The whole process was repeated and again a dried specimen (STD3 and STD4) and one fired sample (STD2B and VAC2B) each were discarded. The steps were repeated, the dried shapes (STD4 and VAC4) were placed down, and the fired shapes were not placed. All steps were repeated one last time. After drying for a minimum of half an hour, all samples were removed from the dryer and placed in a desiccator to cool to room temperature. The break resistance of the specimens was then tested on a TIRA testing machine according to the instructions, at a support spacing of 80 mm. The samples were broken by previously entering their dimensions into the testing machine, at a stress increase rate of 0.15 MPa/s ± 0.015 MPa/s [31].

#### 2.3.2. Determination of Permanent Dimensional Changes Due to Heating to a Specified Temperature

The test was carried out on cylindrical specimens with a height of 60 mm ± 2 mm and a base diameter of 50 mm ± 2 mm, made according to EN 993-10, ISO 2477, and ISO 2478 [6,31]. Three samples each were cut from the block compacted by vibration (STD) and vibration under reduced pressure (VAC), according to the direction of vibration.

The samples, after height measurements (*L_to_*) at ambient temperature (*t*_0_), were placed in a cold furnace, at a minimum distance of 20 mm from each other and a minimum of 50 mm from the furnace walls. The next step was to burn them according to the thermal procedure described in Section 2.3.1. 

Once burning was complete, the samples were removed from the kiln and placed in a desiccator to cool. After cooling, the heights of the samples (*L_t_*) were measured at the same location as before firing, and then, the average height of each sample was calculated, and the percentage of thermal expansion after heating to the highest temperature (*t*) was determined for each sample according to Formula (1).

(1) $$\alpha_{mean}=\frac{L_{t}-L_{t_{0}}}{L_{t_{0}}\left(t-t_{0}\right)},$$

#### 2.3.3. Determination of the Effect of Temperature on the Dimensional Change of the Samples

The test was carried out on 2 specimens, 1 cut from a classically moulded block (STD) and 1 cut from a reduced pressure moulded block (VAC), based on the instructions developed in accordance with EN 993-19. 

The samples were cut in the shape of a cylinder with a diameter of 50 mm ± 0.5 mm and a height of 50 mm ± 0.5 mm, with a 12 to 13 mm diameter hole drilled inside, passing through the entire height of the sample, coaxial to the outer surface of the cylinder. The bases of the cylinder were parallel. The prepared samples had no visible defects (e.g., cracks) on their surfaces. Their dimensions were then measured, i.e., heights at 4 different locations on the sample and diameters—external and internal—at two locations “crosswise”. The average of these measurements was considered as the specimen dimensions.

The thermal expansion analysis consisted of a system mounted in the furnace that sends real-time data to the recording system. The dimensions of the sample were entered into the furnace control programme, air was indicated as the measurement environment, and a temperature of 1500 °C was established. The test was performed according to the described procedure.

#### 2.3.4. Determination of Apparent Density, Open Porosity, and Apparent Specific Gravity

The test was carried out on 10 shapes (STD) and 10 shapes (VAC) cut from compacted blocks. The shapes were regular in shape and easy to dry. They were first dried in a laboratory dryer at 110 ± 5 °C to a constant weight (m_1_). A plastic container was placed in the dry pressure vessel, and a metal grid was placed on the bottom of the container, on which the samples were placed so that they did not touch each other or the walls of the container. The lid was then placed on the vacuum vessel and bolted to the vessel. A rubber hose was used to connect the vacuum pump to the lid valve. The other valves in the vessel were closed. The vacuum pump was turned on. When the PIEZOVAC PV20 pressure gauge read 0 Pa, the pump was switched to lower power, and the pump waited 15 min. The valve connected to the vacuum pump was closed and then turned off. A rubber hose was then connected to one of the valves of the vessel, and the other end was inserted into a distilled water bottle. The valve was gently and slowly opened to draw the water inside the vacuum container. A waiting time of 30 min was made. After half an hour, the valve on the lid of the vessel was slowly opened to equalise the pressure inside the vessel with the pressure outside. The lid was then unscrewed and removed, and the samples were again left for a period of 30 min. The samples thus prepared were weighed on an analytical balance with a basket immersed in distilled water. The system was tared, and then, the individual samples were weighed in water (*m*_2_) and, after being removed and dried with a damp cloth to remove the water from the open pores, were weighed again (*m*_3_). In the calculations, a water density (*ρ_H_*_2*O*_) of 1 g/cm^3^ was assumed.

The results were developed from the formulae for apparent density (2), open porosity (3), and apparent specific gravity (4).

(2) $$\rho_{0}=\frac{m_{1}}{m_{3}-m_{2}}\cdot \rho_{H_{2}O}$$

(3) $$\pi_{a}=\frac{m_{3}-m_{1}}{m_{3}-m_{2}}\cdot 100\%$$

(4) $$A.S.G.=\frac{m_{1}}{m_{1}-m_{2}}$$

#### 2.3.5. Determination of the Effect of Temperature on the Dimensional Change of the Samples

The procedure for preparing samples and performing the test is described in the scheme presented in Section 2.3.3.

#### 2.3.6. Determination of the Total Volume and Pore Size Distribution Using Mercury Porosimetry

The test was carried out on 2 samples prepared from block (STD) and 2 samples prepared from block (VAC) cut with a precision saw, measuring 15 × 15 × 10 mm ± 0.5 mm. The weights of the samples were recorded to the nearest 0.0001 g. Then, they were put aside to cool in a desiccator.

On an AutoPore IV 9500 mercury porosimetry instrument, the analysis parameters were set, and the test was carried out. The tests were carried out in two series: first on samples (STD1) and (VAC1), and then the determination was repeated on samples (STD2) and (VAC2). Empty dilatometers were weighed, and then, samples were transferred to them. The samples were gasified, and then, the dilatometers were filled with mercury to the specified level. The dilatometers with samples and mercury were weighed. The dilatometers with the samples thus prepared were then transferred to the instrument, and the measurement was carried out.

#### 2.3.7. Slag Resistance Test

A slag resistance test was carried out on 2 blocks, (STD) and (VAC), and 4 holes with a diameter (*d*_0_) of 30 mm were drilled into them. The blocks were dried for 4 h in a laboratory dryer at 110 ± 5 °C. The samples were placed in a desiccator for the cooling period. The slags were then prepared according to the previously described material preparation procedure. The slag samples were poured into the drilled holes and covered with ceramic lids. They were then put into the furnace and burnt according to the thermal procedure described in Section 2.3.1.

Once the cycle was completed, the samples were removed from the oven and cooled in air. They were then cut along the centres of the holes drilled in them (the “crucibles”) and then visually assessed, and the slag penetration depths (*d_max_*) were measured. 

The average depth of penetration of the slag was calculated from Equation (5).

(5) $$Slag\;erosion=\frac{d_{max}-d_{0}}{d_{0}}\cdot 100\%$$

**Figure 4:**
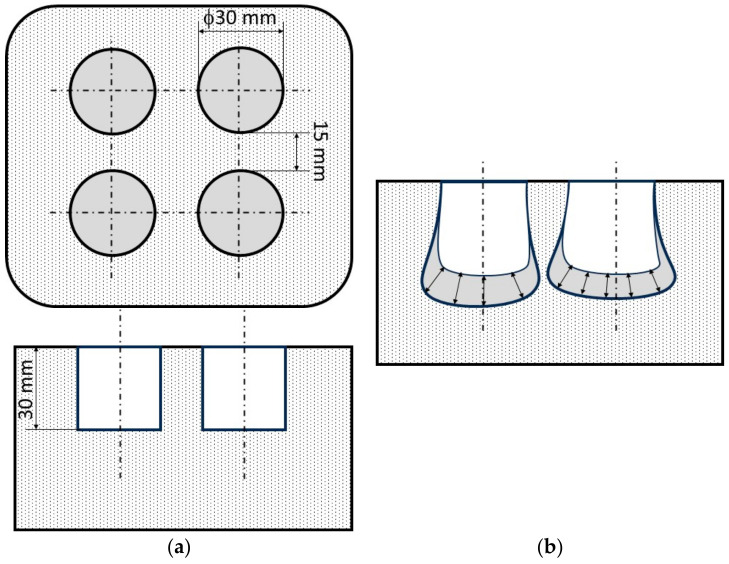
Idea for measuring slag resistance: (**a**) view of the shape before and (**b**) view of the shape after.

The average erosion of the slag was also determined by measuring the area of penetration of the slag in the prepared shapes (STD) and (VAC). The idea of the performed measurement is shown in Figure 4.

## 3. Results

The influence of thermal shock and the resistance of the ceramic fittings to it are the key factors determining the choice of suitable material for products exposed to significant temperature gradients during use. Therefore, the influence of the moulding method on this parameter was investigated first. The results of the tests carried out for dried samples standard moulded at atmospheric pressure (STD numbered 0 to 4) and moulded at reduced pressure (VAC numbered 0 to 4) are shown in Table 1, while Table 2 summarises the results of samples that were fired (STD numbered 1-B to 3-B and VAC numbered 1-B to 3-B). The dimensions of the sample were described by the letters A (width) × B (depth).

**Figure 5:**
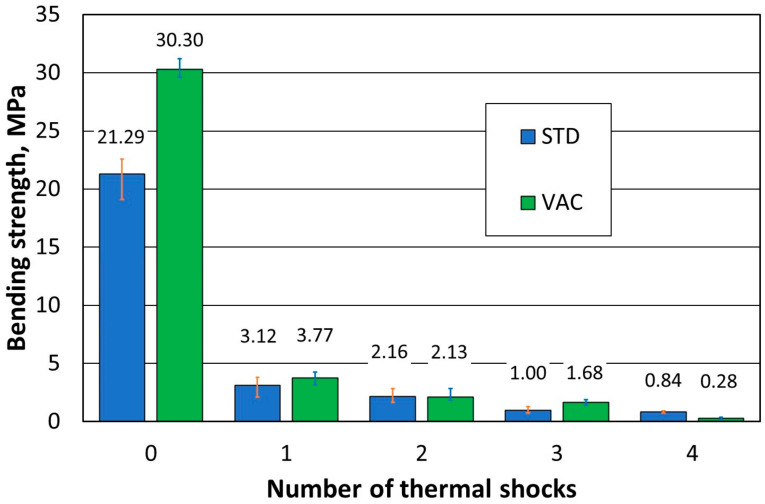
Dependence of flexural strength on the number of thermal shocks in dried specimens.

**Figure 6:**
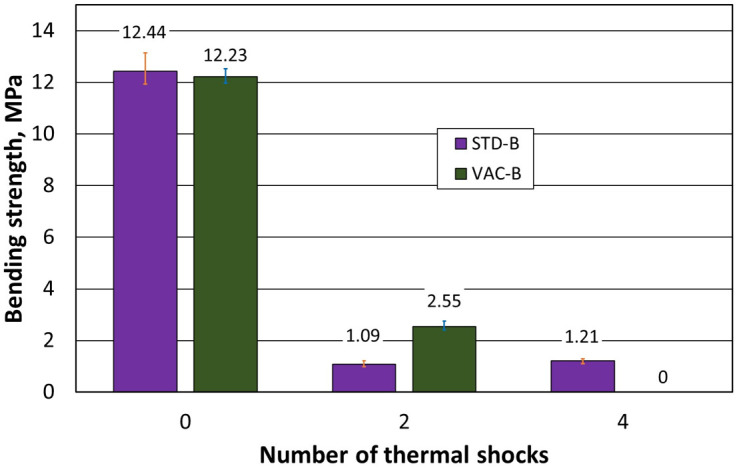
Dependence of flexural strength on the number of thermal shocks in burnt specimens.

Figure 5 shows the results of the effect of the number of thermal shocks for the dried specimens, while Figure 6 shows the results obtained when the specimens were burnt.

Reduced pressure moulding of dried samples shows an increase in strength only for material that has not been subjected to thermal shocks (VAC sample—number of thermal shocks equal to 0). With increasing thermal shocks, no significant effect of the ceramic shape preparation method on the resistance to thermal shocks was found. For the different modes of compaction, a successive decrease in the flexural strength of the samples is noticeable, with increasing thermal shock effects. It was found that mainly the first thermal shock particularly influences the reduction in the flexural strength of the refractory material and causes an approximately seven-fold reduction in the analysed strength.

The initial samples (number of thermal shocks equal to 0) that were fired had lower flexural strengths than the dried specimens. Furthermore, subjecting them to thermal shocks causes a severalfold reduction in their flexural strength. The tests carried out showed the neutral effect of the forming method on the quality of the samples subjected to thermal shocks. 

The next stage of the experimental study was to determine the effect of firing the shaped pieces (symbol (B) in the designation of the samples) that were differently formed in terms of thermal expansion. The tests were performed according to the procedure described earlier. The results of the tests carried out are shown in Table 3 for both samples compacted under standard conditions at atmospheric pressure (STD-B) and shapes compacted under reduced pressure (VAC-B).

**Figure 7:**
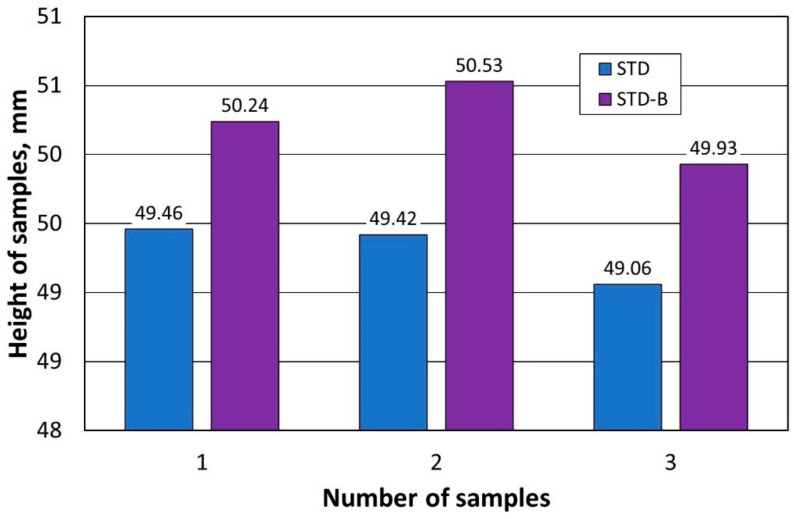
Changes in the dimensions of the samples formed at atmospheric pressure under the firing process.

**Figure 8:**
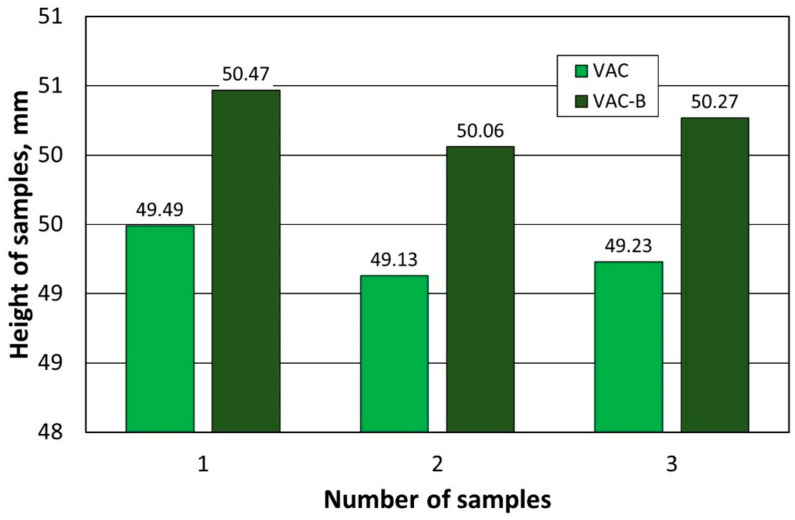
Changes in dimensions of samples formed under reduced pressure under the influence of the firing process.

Figure 7 and Figure 8 summarise the obtained results for comparison.

Analysing the results, it was found that the process of firing the samples increases their dimensions regardless of the moulding method. On the other hand, it can be seen that moulded shapes formed under reduced pressure change their dimensions as a result of firing with a comparable percentage change compared to a large discrepancy in the case of classic moulding. This is probably due to the larger number and larger size of the pores and their uneven distribution in the structure of the shapes that are moulded under atmospheric pressure.

**Figure 9:**
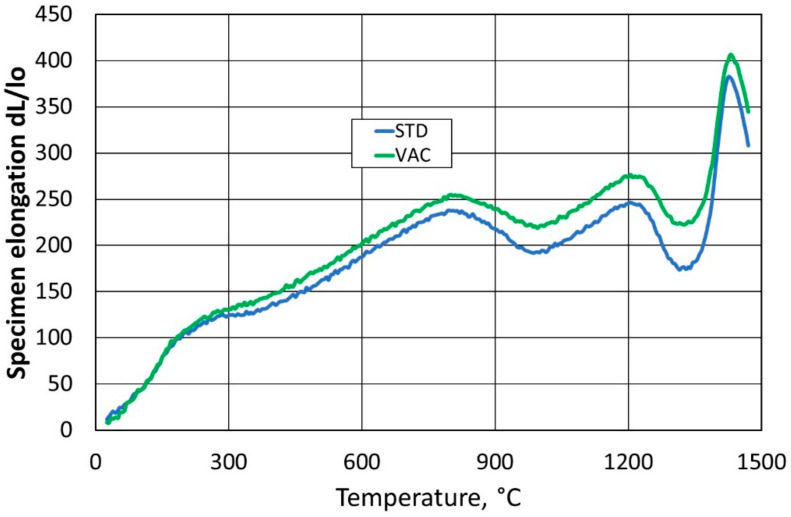
Thermal expansion of the samples (STD) and (VAC).

The effect of the change in the expansion of the samples was also checked as a function of temperature. The results recorded during continuous measurement are shown in Figure 9.

As can be observed in Figure 9, a higher elongation was registered in the vacuum-formed (VAC) specimen. This result is related to the higher density of the material and the assumed more uniform small pore structure. In order to confirm this assumption, three parameters were tested: apparent density, open porosity, and apparent specific gravity (A.S.G). The results were prepared according to the procedure previously described and are presented in Table 4.

The average apparent density value is higher for refractory samples compacted in a reduced-pressure atmosphere (VAC). As expected, vibratory compaction with the creation of a vacuum resulted in a better filling of the moulded lump with ceramic material. Additionally, the creation of a vacuum during the moulding contributed to a noticeable difference in the percentage of open porosity. However, there was no difference in apparent specific gravity between the (VAC) and (STD) samples.

The next stage of the study was to compare the pore structure of the refractory materials. For this, the mercury porosimetry method was used to determine the so-called capillary potential curve. The tests were carried out on two specimens compacted at atmospheric pressure (STD1) and (STD2), and on specimens compacted at reduced pressure (VAC1) and (VAC2).

**Figure 10:**
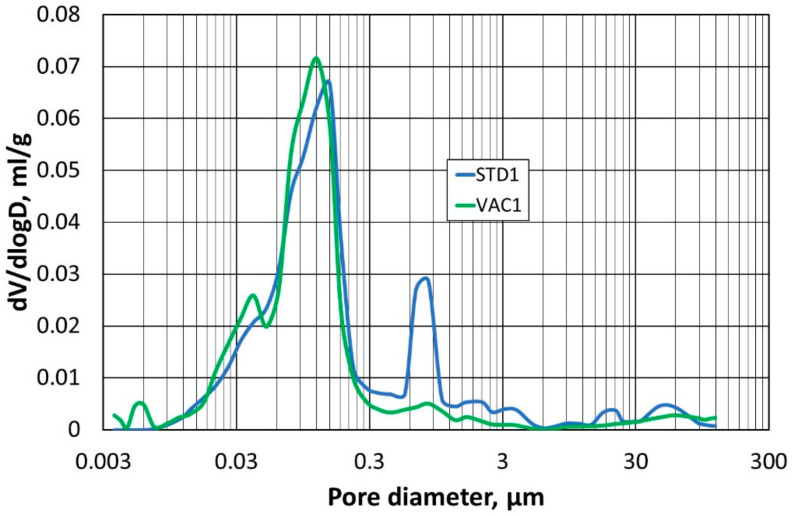
Dependence of pore size distribution on pore diameter for samples (STD1) and (VAC1).

**Figure 11:**
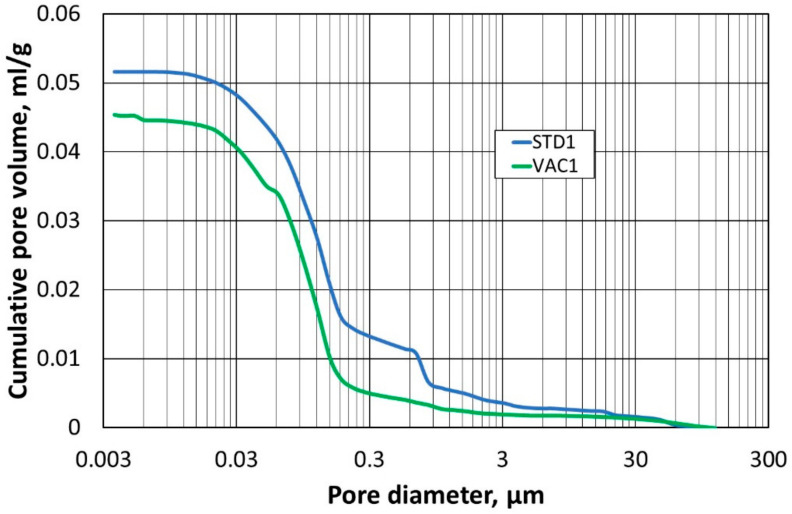
Dependence of total pore volume on pore diameter for samples (STD1) and (VAC1).

**Figure 12:**
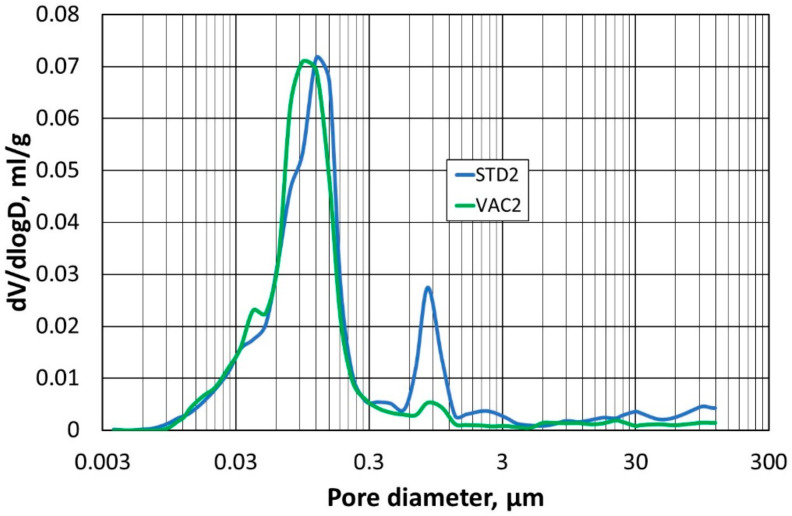
Dependence of pore size distribution on pore diameter for samples (STD2) and (VAC2).

**Figure 13:**
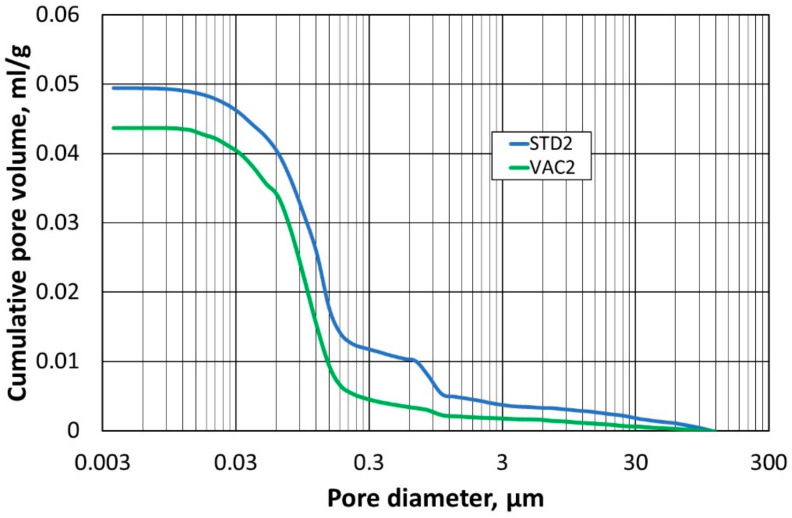
Dependence of total pore volume on pore diameter for samples (STD2) and (VAC2).

In the first step, the samples (STD1) and (VAC1) were compared with each other, and the results of the pore size–diameter distribution are shown in Figure 10, while Figure 11 shows the total pore volume in relation to their diameter. Identical comparisons were made for samples (STD2) and (VAC2), and the corresponding results are shown in Figure 12 and Figure 13.

A test using mercury porosimetry confirmed the assumption that a better compaction of the refractory materials would be obtained in a reduced-pressure atmosphere. The total pore size in the samples (VAC1) and (VAC2) is smaller compared to the samples (STD1) and (STD2). The largest pore size distribution for samples (VAC1), (VAC2), (STD1), and (STD2) is for a pore size of 0.1 µm, but for samples (STD1) and (STD2), another peak for a pore size of 1 µm can be seen in the graph. The absence of an additional peak in the measurements taken (for the two determinations) is indicative of a more homogeneous small pore size in samples (VAC1) and (VAC2), obtained by densification in a reduced-pressure atmosphere.

**Figure 14:**
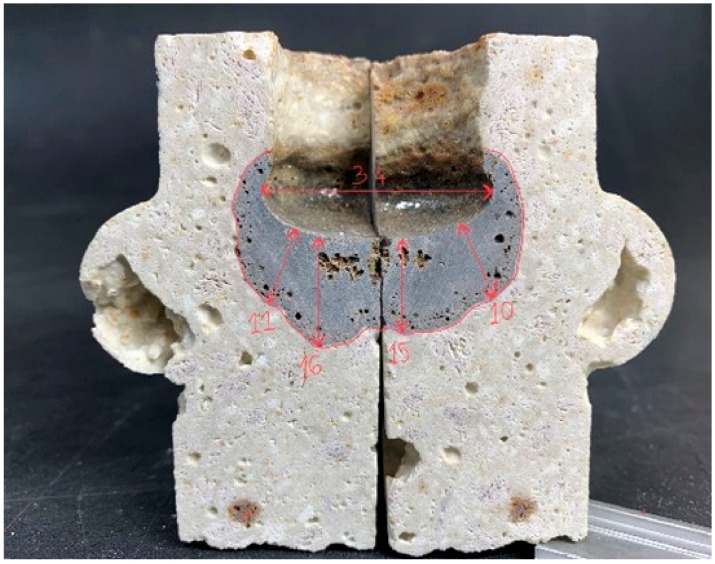
Principle of slag penetration depth measurements [31].

An important criterion for refractories is their resistance to the effects of slag. Slag erosion tests were carried out on suitably prepared shapes compacted under standard atmospheric pressure conditions and reduced by creating a vacuum. Two types of slag were prepared to assess the impact of the resistance of refractory materials to the effects of slags. The average depth of slag penetration was calculated by measuring the depth to which they acted. The principle of measurement is shown in Figure 14.

**Figure 15:**
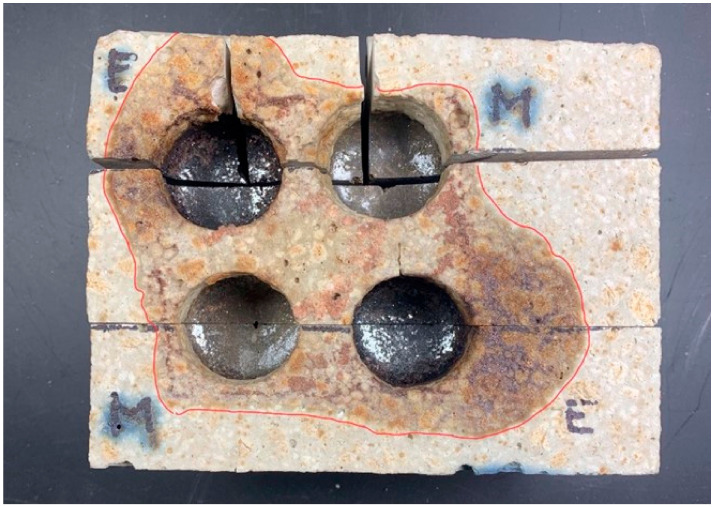
Top view showing the area of action of the slag on the sample surface (STD) [31].

**Figure 16:**
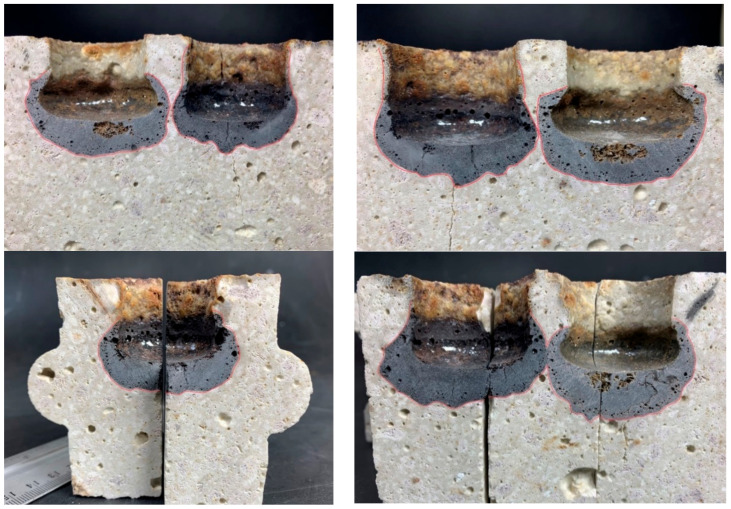
Area of action of slag in the cross-section of sample holes (STD) [31].

**Figure 17:**
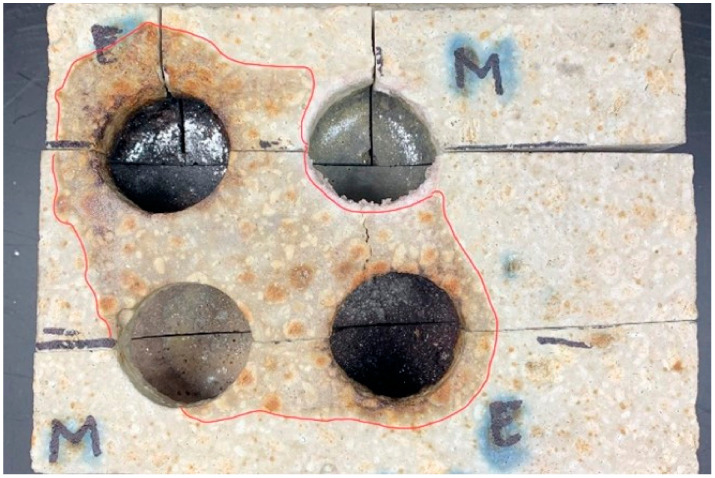
Top view showing the area of action of the slag on the specimen surface (VAC) [31].

**Figure 18:**
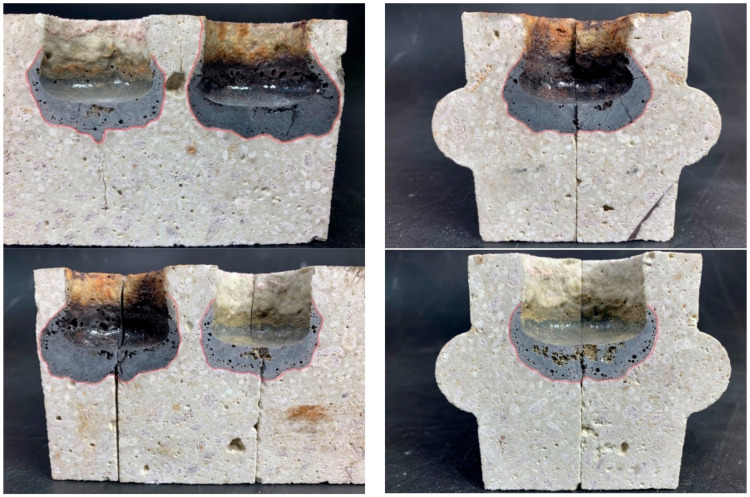
Area of slag action on cross-section of holes in the specimen (VAC) [31].

Figure 15 shows a top view of the slag-treated specimen, and Figure 16 shows a cross-sectional view showing the penetration area in a specimen compacted by the conventional (STD) method. Figure 17, in turn, shows a top view and Figure 18 shows the penetration areas of a sample compacted under a reduced-pressure atmosphere (VAC).

The results of the average depth of penetration of the slag and average slag erosion, relative to the classically moulded block and the reduced pressure moulded block, are shown in Table 5. The study considered the effect of the two types of slag used, i.e., steelworks and the prepared synthetic slag.

The analysis of slag resistance shows the different effects of the type of slag on ceramic material compacted in different ways. Steel mill slag had a smaller effect on the average depth of penetration of the slag of the erosion of a shape compacted at standard atmospheric pressure (STD) in relation to one compacted by creating a vacuum (VAC). In the case of synthetic slag, the opposite relationship was observed. On the other hand, the average erosion of the slag showed a greater effect of synthetic slag on the sections prepared with the different compaction technologies, with steel mill slag having a less effect on the section (VAC) and synthetic slag on the section (STD). The results obtained indicate the need for a custom method of making a specific refractory.

## 4. Conclusions

Broadly performed studies evaluating the spectrum of parameters influencing the method of preparation of refractory materials allow several observations to be made.

The vibratory compaction of refractories using reduced pressure increases the bending strength of dried samples. No such effect was found for fired samples. No beneficial effect was found on the thermal shock resistance of the refractory tested, with regard to the method of compaction or the final preparation of the material (drying and firing).

For both methods, firing increases the dimensions of the test specimens. However, firing the compacted under reduced pressure has a beneficial effect on stabilising their dimensions.

For vibrationally compacted refractory material under reduced pressure, it was found that there was a greater linear expansion due to the higher apparent density of the material. This is due to a reduction in the size of the open pores, which is the result of a better distribution, i.e., packing, of the material in the mould.

The statements mentioned above were also confirmed using mercury porosimetry tests of the refractory materials tested, which were performed in different ways. The tests carried out indicate the occurrence of fewer pores of a small size in a mould vibrationally compacted under reduced pressure.

The better compaction of the refractory material, combined with the smaller number and size of pores present, is not insignificant for the next parameter assessed, which is the resistance to slag. Through testing, it was found that material compacted under reduced pressure showed a better resistance to steel mill slag if the average slag erosion was used as an assessment criterion. In the case of synthetic slag, the opposite results were obtained. This may indicate the need to prepare the refractory in a specific way, depending on its operating conditions.

The proposed method of forming refractories In a reduced-pressure atmosphere is an innovative method for improving and stabilising the parameters of the finished refractories.

The presented methodology for the evaluation of refractory materials is a universal tool, enabling their appropriate selection depending on the character/scope of foundry operation at the stage of designing the lining of melting furnaces in terms of construction, modernisation, overhaul, or repair.

## Figures and Tables

**Table 1:** Bending strength results for dried samples.

Name	Number of Thermal Shocks	A (mm)	B (mm)	Bending Strength (MPa)
STD0	0	20.45	20.03	21.29
STD1	1	19.80	19.79	3.12
STD2	2	20.18	20.09	2.16
STD3	3	20.33	20.25	1.00
STD4	4	19.51	19.98	0.84
VAC0	0	20.80	21.05	30.03
VAC1	1	20.78	20.78	3.77
VAC2	2	20.65	20.73	2.13
VAC3	3	20.72	21.10	1.68
VAC4	4	20.71	20.55	0.28

**Table 2:** Bending strength results for fired samples.

Name	Number of Thermal Shocks	A (mm)	B (mm)	Bending Strength (MPa)
STD1–B	0	21.09	20.85	12.44
STD2–B	2	20.32	20.85	1.09
STD3–B	4	20.21	20.33	1.21
VAC1–B	0	20.54	20.78	12.23
VAC2–B	2	20.91	20.93	2.55
VAC3–B	4	21.00	20.33	-

**Table 3:** Results of determination of permanent dimensional change due to heating to a specific temperature [31].

Nr	Height of Sample Before Testing (mm)	Average (mm)	Sample Height After Testing (mm)	Average (mm)	Result (%)
Samples STD and STD–B					
1	49.19	49.46	49.76	50.24	+1.57
	49.42		50.34		
	49.70		50.72		
	49.53		50.12		
2	49.53	49.42	50.75	50.53	+2.25
	49.50		50.08		
	49.29		50.27		
	49.34		51.00		
3	49.10	49.06	50.15	49.93	+1.78
	49.04		49.88		
	49.02		49.84		
	49.06		49.85		
	49.29		49.96		
Samples VAC and VAC–B					
1	49.33	49.49	50.09	50.47	+1.99
	49.57		50.81		
	49.62		50.62		
	49.42		50.35		
2	49.10	49.14	50.00	50.06	+1.88
	49.09		49.92		
	49.15		49.91		
	49.20		50.40		
3	49.50	49.23	50.40	50.27	+2.13
	49.35		50.70		
	48.94		49.96		
	49.11		50.03		

**Table 4:** Apparent density, open porosity, and A.S.G. test results for VAC and STD samples.

Name	Average Density Apparent (g/cm^3^)	Average Open Porosity (%)	Apparent Specific Gravity (A.S.G.)
STD	2.36	15.03	2.78
VAC	2.41	13.13	2.77

**Table 5:** Results of average slag penetration depth and average slag erosion for both slags for (STD) and (VAC) blocks [31].

Slag	Average Depth of Slag Penetration (mm)	Average Cinder Erosion (%)
STD		
Slag from the steelworks	8.73	14.17
Synthetic slag	12.31	22.50
VAC		
Slag from the steelworks	10.17	11.11
Synthetic slag	10.77	27.78

## Data Availability

The data that support the findings of this study are available from the corresponding authors (M.B.) upon reasonable request.
